# Changes in lipid indices and body composition one year after laparoscopic gastrectomy: a prospective study

**DOI:** 10.1186/s12944-018-0729-1

**Published:** 2018-05-11

**Authors:** Soo Jin Lee, Ji Young Kim, Tae Kyung Ha, Yun Young Choi

**Affiliations:** 1 Department of Nuclear Medicine, Hanyang University Medical Center, 222-1 Wangsimni-ro, Seongdong-gu, Seoul, 133-792 South Korea; 2 Department of Nuclear Medicine, Hanyang University College of Medicine, 222-1 Wangsimni-ro, Seongdong-gu, Seoul 133-792 South Korea; 3 Department of Surgery, Hanyang University College of Medicine, 222-1 Wangsimni-ro, Seongdong-gu, Seoul 133-792 South Korea

**Keywords:** Lipids, Body composition, Weight loss, Gastrectomy

## Abstract

**Background:**

The purpose of this prospective study was to investigate changes in lipid indices associated with whole body composition during 1 year of follow-up after laparoscopic gastrectomy.

**Methods:**

Thirty-seven patients with benign and malignant gastric neoplasm who underwent laparoscopic gastrectomy were prospectively enrolled. None of the patients were treated with adjuvant chemotherapy. Lipid indices and body composition were measured preoperatively and at six and 12 months after laparoscopic gastrectomy. Lipid indices included total cholesterol (TC), triglyceride (TG), low-density lipoprotein cholesterol (LDL-C) and high-density lipoprotein cholesterol (HDL-C). Body weight, fat and lean body mass (LBM) were measured by dual-energy X-ray absorptiometry and the change in fat and LBM in the trunk, arms and legs was compared.

**Results:**

Body weight significantly decreased from 63.0 ± 11.1 kg preoperatively to 56.8 ± 10.6 kg 12 months after laparoscopic gastrectomy, with a mean of 7.1% (4.6 kg) weight loss. Fat and LBM loss contributed 68.4% (3.1 kg) and 30.1% (1.4 kg) of the total weight loss, respectively. In both the non-obese and obese groups, body weight, fat and LBM did not change significantly between 6 months and 12 months after gastrectomy. TC and LDL-C levels significantly decreased during the first six-month period and HDL-C significantly increased until 12 months after gastrectomy in the non-obese group. In the obese group, the degree of reduction in fat mass was significantly higher and the LBM/weight ratio significantly increased compared with the non-obese group. However, there was no significant change in lipid indices in the obese group. The TG level was significantly correlated with fat, especially with trunk fat.

**Conclusion:**

Gastrectomy resulted in improved lipid indices and a reduction in body weight, fat and LBM. The HDL-C significantly increased in the non-obese group for 1 year after gastrectomy and the reduction of TG level was positively correlated with fat, especially with trunk fat (IRB No. 2015-04-026. Registered 4 May 2015).

## Background

Gastrectomy is used as the standard treatment for gastric neoplasm, regardless of tumor depth. Associated outcomes include significant improvements in lipid indices [1, 2] and results in 10 – 20% weight loss within 3 months after surgery [3]. In obese patients, sleeve gastrectomy has been well-established as an effective intervention to reduce body weight and often improves lipid indices [4]. After gastrectomy, patients with early gastric cancer experienced decreases in low-density lipoprotein cholesterol (LDL-C) while high-density lipoprotein cholesterol (HDL-C) increased. Consequently, these lipid changes were followed by a reduction in cardiovascular risk [1, 5].

Fat loss is the main cause of weight loss, while loss of lean body mass (LBM) contributed minimally [6, 7]. Weight loss and subsequent changes in body composition are expected because the volume of digestive organ available for nutrient absorption is reduced after gastrectomy. Recent studies reported that the cause of this was attributable to nutritional or metabolic disturbances that result from impaired food intake, transit time and malabsorption [7, 8]. In addition, decreases in gastrointestinal hormone levels can have an effect [9]. The amount of weight loss depends upon gastrectomy and anastomosis type [10, 11].

It is not still fully understood how changes in body composition following gastrectomy are related to lipid indices. Studies of weight loss after gastrectomy have mainly focused on fat loss, and few studies have assessed associations with LBM, although the effects of gastrectomy on LBM were observed throughout the body, including the trunk, arms and legs [10]. Changes in LBM should be monitored since the rapid loss of muscle mass, regardless of the presence of cancer, is associated with higher mortality [12].

In our study, we prospectively investigated the change in body composition and lipid indices, and the association between these differences in patients with gastric neoplasms over 1 year of follow-up after laparoscopic gastrectomy. We compared the change in fat and LBM in body compartments, including the trunk, arms and legs. In addition, we observed the difference in these factors between non-obese and obese groups.

## Methods

### Patients and study design

This was a prospective study, approved by the Institutional Review Board of our medical center (IRB No. 2015-04-026), and informed consent was submitted by all subjects when they were enrolled. Thirty-seven patients with benign or malignant gastric neoplasms who underwent gastrectomy were enrolled and followed for 12 months between May 2015 and December 2016. The patients underwent body composition analysis and lipid indices preoperatively and 6 months after laparoscopic gastrectomy. Among these, 33 patients continued in the study until 12 months after gastrectomy. Patients with advanced gastric cancer, who were treated with postoperative chemotherapy or were malnourished, were excluded.

All patients underwent curative laparoscopic gastrectomy performed by a single surgeon (TK Ha). Surgical procedures were as follows: (1) wedge resection, subtotal or total gastrectomy were performed with or without lymph node dissection, according to the second edition of the Japanese Classification of Gastric Cancer; (2) R0 resection was performed; (3) laparoscopic gastrectomy was performed; and (4) gastroduodenal anastomosis or duodenal bypass, including Roux-en-Y or gastrojejunostomy.

Blood samples were collected following an overnight fast to measure total cholesterol (TC), triglyceride (TG), LDL-C and HDL-C before gastrectomy as well as at six and twelve months after the gastrectomy.

Body composition measurements were analyzed by dual-energy X-ray absorptiometry (QDR 4500, Hologic, Waltham, MA, USA), which provides body weight, bone mineral composition (BMC), LBM, and fat. Body mass index (BMI) was calculated as weight (kilograms, kg) divided by height (meters^2^). The distribution of fat was measured in the trunk and both arms and legs. Body composition was measured within 1 week before surgery and at six and twelve months after gastrectomy. We assessed patient nutrition before and after gastrectomy. Patients followed a diet and were analyzed for caloric intake by a hospital nutritionist.

### Statistical analyses

All data are expressed as mean ± standard deviation (SD). A paired *t*-test was used to assess the difference in each variable before surgery and at six and twelve months after surgery. The Mann-Whitney test was used to compare variables between non-obese and obese groups. Statistical analyses were performed using commercial software packages (SPSS version 19, IBM, Chicago, IL; MedCalc version 14, MedCalc Software bvba, Ostend, Belgium), and *P*-values less than 0.05 were regarded as statistically significant.

## Results

Patient characteristics are summarized in Table 1. Thirty-seven patients, consisting of 24 men and 13 women, were enrolled in this prospective study. There were no complications and patient eating progressed favorably after surgery and in consultation with a hospital nutritionist.

**Table 1:** Patient characteristics

	Number	%
Number of patients	37	
Age (years)	56.0 ± 11.0	
Male: Female	24: 13	63.9: 35.1
Height (cm)	165.5 ± 8.8	
Body weight (kg)	63.4 ± 10.9	
BMI (kg/m^2^)	23.1 ± 3.4	
Non-obesity (< 25 kg/m^2^)	26	
Obesity (≥ 25 kg/m^2^)	11	
Tumor stage		
I	37	100
Tumor depth		
T1	29	78.4
T2	8	21.6
Extent of resection		
Wedge	7	18.9
Subtotal	28	75.7
Total	2	5.4
Lymph node dissection		
None	7	18.9
D1	6	16.2
D2	24	64.9
Anastomosis		
None	6	16.2
Billroth I	1	2.7
Billroth II	1	2.7
Roux-en-Y	29	78.4

*BMI* body mass index

### Body composition and lipid indices 6 months after gastrectomy

Preoperative BMI, body weight, fat, LBM, BMC and lipid indices were compared with those measured at 6 months and 12 months after gastrectomy (Table 2). During the first 6 months, the mean BMI decreased from 23.1 ± 3.4 kg/m^2^ to 21.4 ± 2.8 kg/m^2^ (− 7.4%) and mean body weight decreased from 63.4 ± 10.9 kg to 58.8 ± 9.8 kg (*P* < 0.001, for each) with a mean of 7.3% weight loss. In addition, LBM and fat decreased by 3.3% and 20.8% (*P* < 0.001, for each). Preoperative BMC was significantly higher than at 6 months postoperatively, although the difference was small, 2.1% (*P* = 0.004). Fat loss was a major cause of weight loss after gastrectomy. The change in fat distribution in the trunk was the largest, followed by changes in the arms and legs.

**Table 2:** The changes in body composition and lipid indices at baseline, 6 months and 12 months after surgery

	Baseline	Δ 6 months	Δ 12 months	^*^P value	^**^P value	^*§*^*P* value
BMI (kg/m^2^)	23.1 ± 3.4	−1.72 ± 1.7	−1.75 ± 2.1	< 0.001	0.821	0.030
Body weight (kg)	63.4 ± 10.9	−4.60 ± 4.6	−4.58 ± 5.3	< 0.001	0.992	< 0.001
BMC (kg)	2.21 ± 0.4	− 0.046 ± 0.091	−0.068 ± 0.11	0.004	0.298	0.002
LBM (kg)	46.7 ± 9.2	− 1.55 ± 2.4	−3.05 ± 2.8	< 0.001	0.531	0.002
Fat (kg)	14.4 ± 5.3	− 3.00 ± 3.7	−3.13 ± 4.6	< 0.001	0.527	< 0.001
Both arms	2.26 ± 1.0	− 0.53 ± 0.6	− 0.64 ± 0.8	< 0.001	0.125	< 0.001
Trunk	7.28 ± 3.1	− 1.82 ± 2.4	−1.78 ± 2.9	< 0.001	0.878	0.002
Both legs	3.95 ± 1.5	− 0.62 ± 0.89	−0.95 ± 1.36	< 0.001	0.960	0.021
Hb (g/dL)	13.2 ± 2.0	−0.024 ± 1.9	−0.20 ± 2.0	0.761	0.461	0.646
Protein (g/dL)	6.8 ± 0.7	−0.29 ± 0.8	−0.21 ± 0.8	0.055	0.186	0.241
TC (mg/dL)	185.6 ± 35.4	− 16.6 ± 23.1	−10.5 ± 23.7	0.011	0.350	0.137
TG (mg/dL)	125.4 ± 85.4	− 14.5 ± 39.8	− 17.4 ± 34.2	0.367	0.274	0.514
HDL-C (mg/dL)	42.4 ± 10.4	4.2 ± 9.4	9.3 ± 9.9	0.007	0.245	0.003
LDL-C (mg/dL)	110.1 ± 33.5	−14.0 ± 36	−6.2 ± 30	0.015	0.074	0.361

Date are presented as mean ± SD, *BMI* body mass index, *BMC* bone mineral composition, *LBM* lean body mass, *TC* total cholesterol, *TG* total glyceride, *HDL-C* high-density lipoprotein Cholesterol, *LDL-C* low-density lipoprotein

^*^Paired *t*-test between baseline and 6 months; ^*^Paired *t*-test between 6 months and 12 months; ^*§*^Paired *t* test between baseline and 12 months

The TC (− 8.9%) and LDL-C (− 12.7%) significantly decreased 6 months after surgery. The mean level of HDL-C significantly increased from 42.4 ± 10.4 mL/dL to 46.6 ± 10.3 mL/dL (+ 9.9%). However, TG values were not significantly different preoperatively and 6 months after surgery. There was no notable change in mean hemoglobin and protein values.

Among 37 patients, 33 patients (24 non-obese and 9 obese) were followed for 12 months after surgery. All body composition parameters including BMI, body weight, fat, LBM and BMC, and HDL-C differed significantly between baseline and 12 months after gastrectomy.

### Group comparisons

Patients were classified into two groups; non-obese (BMI < 25 kg/m^2^) and obese (BMI ≥ 25 kg/m^2^). There were 26 patients (70.3%) who were non-obese and 11 patients (29.7%) were obese. In Table 3, the mean values of preoperative BMI, body weight and fat in the obese group were significantly higher than those in the non-obese group. The mean fat values were 12.0 ± 4.0 kg for the non-obese group and 20.2 ± 3.5 kg for the obese group. The preoperative mean BMC, LBM, lipid parameters (TC, TG, HDL-C and LDL-C) and protein levels did not significantly differ between the two groups.

**Table 3:** Preoperative body composition and lipid indices between non-obese and obese groups

	Non-obesity (*n* = 26)	Obesity (*n* = 11)	*P value
Age (years)	54.5 ± 9.6	61.7 ± 12.5	0.379
Male: Female	17: 9	7: 4	
Resection type			
Wedge/subtotal/total	5/21/0	2/7/2	
BMI (kg/m^2^)	21.3 ± 2.1	27.4 ± 1.7	< 0.001
Body weight (kg)	58.9 ± 7.8	73.8 ± 10.3	< 0.001
LBM (kg)	44.8 ± 7.9	51.2 ± 10.7	0.053
BMC (kg)	2.12 ± 0.4	2.40 ± 0.4	0.066
Fat (kg)	12.0 ± 4.0	20.2 ± 3.5	< 0.001
TC (mg/dL)	183.8 ± 35.5	190.0 ± 36.6	0.207
TG (mg/dL)	126.9 ± 84.3	121.7 ± 91.8	0.711
HDL-C (mg/dL)	42.0 ± 11.1	43.2 ± 8.8	0.065
LDL-C (mg/dL)	110.0 ± 30.6	110.4 ± 41.1	0.728

Date are presented as mean ± SD, *BMI* body mass index, *LBM* lean body mass, *BMC* bone mineral composition, *TC* total cholesterol, *TG* total glyceride, *HDL-C* high-density lipoprotein cholesterol, *LDL-C* low-density lipoprotein cholesterol

*Mann-Whitney test

Table 4 shows the changes in lipid indices and body composition within groups and between groups. Six months after surgery, the non-obese group had significantly lower BMI, body weight, BMC, LBM and fat mass. Their TC markedly decreased from 183.8 ± 35.5 mg/dL to 168.8 ± 31.7 mg/dL with a mean decrease of 8.2%, and HDL-C increased from 42.0 ± 11.1 mg/dL to 47.4 ± 11.0 mg/dL with an increase of 12.9%. However, the mean TG and LDL-C did not significantly change. In the obese group, BMI, body weight, LBM and fat mass significantly decreased after surgery (− 12.8%, − 12.5%, − 5.2% and − 32.3%). Among 11 patients, 7 patients (7/11, 63.6%) had a BMI below 25 kg/m^2^. However, there were no differences in BMC and lipid indices after surgery, although the mean preoperative TC, TG and LDL-C were higher.

**Table 4:** Changes in body composition and lipid indices between non-obese and obese groups after gastrectomy

	Non-obesity (n = 26)						Obesity (n = 11)					
	Baseline	Δ 6 months	Δ 12 months	*P	**P	^*§*^ *P*	Baseline	Δ 6 months	Δ 12 months	*P	**P	^*§*^ *P*
BMI (kg/m^2^)	21.3 ± 2.1	−0.96 ± 1.0	− 0.97 ± 0.9	< 0.001	0.617	< 0.001	27.4 ± 1.7	− 3.51 ± 1.8	−3.81 ± 2.8	0.003	0.953	0.015
Body weight (kg)	58.9 ± 7.8	−2.64 ± 2.9	− 2.65 ± 2.5	< 0.001	0.627	< 0.001	73.8 ± 10.3	− 9.22 ± 4.7	− 9.72 ± 7.3	0.003	0.953	0.015
LBM (kg)	44.8 ± 7.9	− 1.09 ± 2.2	− 0.95 ± 2.3	0.018	0.424	0.037	51.2 ± 10.7	− 2.64 ± 2.7	−2.53 ± 2.4	0.016	0.441	0.015
BMC (kg)	2.12 ± 0.4	− 0.043 ± 0.063	− 0.064 ± 0.083	0.004	0.230	0.003	2.40 ± 0.4	− 0.054 ± 0.14	−0.077 ± 0.18	0.091	0.678	0.260
Fat (kg)	12.0 ± 4.0	− 1.50 ± 2.5	− 1.64 ± 2.8	0.008	0.841	0.018	20.2 ± 3.5	− 6.53 ± 3.9	−7.11 ± 6.2	0.003	0.953	0.021
TC (mg/dL)	183.8 ± 35.5	− 15.1 ± 35.3	−7.78 ± 22.5	0.038	0.037	0.354	190.0 ± 36.6	− 20.7 ± 51.7	−18.8 ± 45.3	0.285	0.779	0.612
TG (mg/dL)	126.9 ± 84.3	− 16.7 ± 95.3	−19.9 ± 86.1	0.970	0.558	0.449	121.7 ± 91.8	− 9.55 ± 116	− 10.6 ± 147	0.929	1.000	0.514
HDL-C (mg/dL)	42.0 ± 11.1	5.42 ± 9.5	11.0 ± 9.5	0.007	< 0.001	< 0.001	43.2 ± 8.8	1.36 ± 8.7	5.00 ± 10.3	0.448	0.400	0.214
LDL-C (mg/dL)	110.0 ± 30.6	− 12.9 ± 33.5	−4.96 ± 26.4	0.069	0.013	0.339	110.4 ± 41.1	−16.5 ± 43.1	−9.44 ± 40.2	0.306	0.401	1.000

Date are presented as mean ± SD, *BMI* body mass index, *LBM* lean body mass, *BMC* bone mineral composition, *TC* total cholesterol, *TG* total glyceride, *HDL-C* high-density lipoprotein cholesterol, *LDL-C* low-density lipoprotein cholesterol

* Paired *t* test between baseline and 6 months; ** Paired *t* test between 6 months and 12 months; ^*§*^ Paired *t* test between baseline and 12 months

Between 6 months and 12 months after gastrectomy, all body composition parameters did not significantly change in either group. In the non-obese group, TC and LDL-C significantly increased between 6 months and 12 months after gastrectomy, however there was no noticeable change between baseline and 12 months. During the 12 months of follow-up, both groups had considerably lower BMI, body weight, LBM and fat mass, compared with preoperative values. During the 12 months after gastrectomy, the mean weight loss was 2.65 kg for the non-obese group and 9.72 kg for the obese group. The mean fat loss was 1.64 kg for the non-obese group and 7.11 kg for the obese group. The mean BMC loss was 0.07 kg for the non-obese group and 0.08 kg for the obese group. The non-obesity group had significantly lower BMC and higher HDL-C value.

### Fat and LBM distribution changes in the trunk and limbs 12 months after gastrectomy

The changes (%) in fat and LBM were compared between the two groups using independent *t-*tests (Table 5). Changes in fat were significantly greater in the obese group and significant changes were found in both trunk and extremities. The change in LBM values in extremities was significantly greater in the obese group.

**Table 5:** Percent change of fat and LBM in non-obese and obese groups

	Non-obesity (*n* = 26)		Obesity (*n* = 11)			
	6 months	12 months	6 months	12 months	*P	**P
Total fat	- 10.0 ± 24.1	− 10.4 ± 24.9	- 31.1 ± 17.0	- 33.6 ± 29.0	0.012	0.030
Trunk	- 12.8 ± 23.6	- 13.8 ± 32.1	- 36.5 ± 19.9	- 34.5 ± 35.4	0.019	0.118
Extremities	- 7.7 ± 27.6	- 5.9 ± 27.6	- 26.6 ± 16.7	- 35.1 ± 24.2	0.042	0.009
Total LBM	- 2.1 ± 5.8	- 1.7 ± 5.9	- 5.5 ± 5.3	- 5.6 ± 5.2	0.102	0.089
Trunk	- 2.2 ± 6.7	- 1.8 ± 8.1	- 3.8 ± 5.0	- 2.9 ± 6.5	0.481	0.720
Extremities	- 2.5 ± 8.4	- 2.1 ± 7.3	- 7.1 ± 10.1	- 7.9 ± 6.9	0.165	0.047

* Independent *t* test between two groups for first 6 months; ** Independent *t* test between two groups for 12 months

**Fig. 1:**
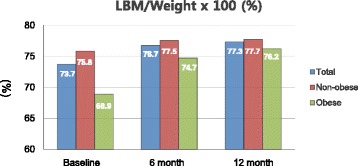
Ratio of lean body mass (LBM)/weight (× 100)

Figure 1 shows the change of LBM/weight ratio in non-obese and obese groups up to 12 months after gastrectomy. The LBM/weight ratio of the non-obese group did not change significantly during the 12 months after gastrectomy. The LBM/weight ratio of obese groups significantly increased 6 months after gastrectomy, but did not change considerably thereafter.

### Body composition and lipid indices

Body composition parameters including BMI, body weight, fat and LBM were compared with the lipid indices of TC, TG, HDL-C and LDL-C (Table 6). The TC, HDL-C and LDL-C did not correlate with body composition parameters. The TG was the only significantly different value related to the fat, especially trunk fat. Positive moderate correlations between fat and TG (*r* = 0.520, *P* = 0.002), and trunk fat and TG (*r* = 0.588, *P* < 0.001) at 12 months after gastrectomy were observed.

**Table 6:** Correlation between body composition and lipid indices (correlation values/ *P* value)

	BMI	Body weight	Fat	LBM	Trunk fat	Trunk LBM
Baseline						
TC (mg/dL)	0.060/0.727	0.123/0.474	0.176/0.304	0.048/0.783	0.213/0.212	0.073/0.671
TG (mg/dL)	0.086/0.613	− 0.006/0.973	0.133/0.431	−0.076/0.653	0.150/0.374	−0.114/0.501
HDL-C (mg/dL)	−0.075/0.657	0.108/0.525	0.158/0.352	0.038/0.824	0.172/0.310	0.084/0.622
LDL-C (mg/dL)	0.086/0.614	0.108/0.526	−0.009/0.957	0.123/0.437	−0.013/0.940	0.114/0.501
6 months						
TC (mg/dL)	0.164/0.332	0.129/0.446	0.106/0.534	0.108/0.525	0.241/0.151	0.087/0.609
TG (mg/dL)	0.246/0.143	0.241/0.151	0.353/0.032	0.127/0.455	0.442/0.006	0.090/0.597
HDL-C (mg/dL)	−0.210/0.211	−0.094/0.580	−0.039/0.819	− 0.086/0.611	−0.059/0.727	− 0.090/0.597
LDL-C (mg/dL)	0.216/0.200	0.067/0.692	0.047/0.782	0.067/0.695	0.199/0.237	0.048/0.777
12 months						
TC (mg/dL)	0.115/0.524	0.045/0.802	0.229/0.199	−0.034/0.852	0.201/0.262	0.003/0.986
TG (mg/dL)	0.470/0.006	0.453/0.008	0.520/0.002	0.315/0.074	0.588/< 0.001	0.322/0.068
HDL-C (mg/dL)	−0.194/0.279	−0.189/0.292	− 0.121/0.503	−0.166/0.356	− 0.137/0.448	−0.177/0.325
LDL-C (mg/dL)	0.196/0.275	0.217/0.225	0.217/0.224	0.166/0.357	0.260/0.145	0.177/0.325

*BMI* body mass index, *TC* total cholesterol, *TG* total glyceride, *HDL-C* high-density lipoprotein cholesterol, *LDL-C* low-density lipoprotein cholesterol

## Discussion

Although previous studies have reported statistically significant differences in the change of BMI, weight, fat and lipid indices after gastrectomy, there are currently only a limited number of studies that establish their association with whole body composition. In this prospective study, we observed a significant difference in lipid indices and whole body composition for 1 year after laparoscopic gastrectomy. Gastrectomy resulted in a reduction in body weight, fat, LBM and improved lipid indices (TC, LDL-C and HDL-C) 6 months after gastrectomy. The change in HDL-C continued until 12 months after gastrectomy, however TG and LDL-C did not significantly change between baseline and 12 months. The reduction of fat mass occurred in each compartment of the arm, trunk and legs 6 months after gastrectomy. TG level was significantly correlated with fat, especially trunk fat. The degree of reduction in fat mass of the obese group was significantly higher and the LBM/weight ratio significantly increased during 12 months compared with those of the non-obese group. However, there was no significant change in lipid indices in the obesity group.

Previous studies have reported that gastrectomy types affect the extent of weight loss, with the least amount of weight loss observed after laparoscopic gastrectomy [6, 7, 10]. Fat loss is the primary driver of weight loss in these patients. Fat loss occurs secondary to a reduction in gastric size, impaired oral intake, fat malabsorption due to rapid intestinal transit and changes in gastrointestinal hormone levels [10, 11, 13]. In our study, fat loss occurred throughout the body, including the trunk and both arms and legs in the initial 6 months postoperatively. Thereafter fat change was not significant. Fat mass in the arms significantly decreased and this finding was consistent with that of a previous study [6].

Loss of muscle mass occurs in the first month after laparoscopic surgery, and recovers to the preoperative state afterward [10]. The perioperative muscle loss may be due to poor nutrition and lack of physical activity, and can normalize after patients returned to healthy eating and physical activity. A previous study showed that bariatric surgery in morbidly obese patients with an average BMI of 48.7 kg/m^2^ resulted in a mean weight loss of 50.9 kg, including 75.2% of fat mass and 24.8% of LBM [14]. In our study, in this period, the non-obese group had a mean weight loss of 2.65 kg and LBM loss of 1.0 kg (37.7% of weight loss), and the obese group had a mean weight loss of 9.7 kg and LBM loss of 2.5 kg (25.8% of weight loss). The LBM of the non-obese group comprised a relatively higher proportion of weight loss after gastrectomy. In general, skeletal muscle loss had no impact on survival in patients with gastric cancer; however, rapid loss of muscle mass in older people was associated with higher mortality [15]. Therefore, LBM loss should be prevented in older patients with a nutritionally balanced diet and physical activity should be introduced earlier in the postoperative period.

The BMC in the non-obese group decreased in the first 6 months and this might indirectly signify a decrease in bone mineral density. Bariatric surgery such as Roux-en-Y gastric bypass is known to cause moderate malabsorption in which there is an increase in bone resorption and a reduction in bone mineral density in the first year after surgery [16]. This can be prevented by supplying enough vitamin D or calcium and encouraging early exercise after gastrectomy.

Gastrectomy is generally associated with improved lipid indices regardless of obesity. In this study, the levels of TC and LDL-C significantly decreased while HDL-C increased during 6 months after gastrectomy. It is noteworthy that HDL-C significantly increased until 12 months after surgery, although other indices did not change. In a previous study, we reported that lipid indices including TC, TG, LDL-C and HDL-C significantly improved 1 year after gastrectomy in patients with early gastric cancer and suggested that a reduction in body weight and fat might improve lipid profiles and prevent atherosclerotic changes [7]. There was some difference between our studies, and this may be due to our small study population. We previously reported a weak negative correlation between change in visceral fat area and HDL-C level. In this study, there was no meaningful relationship between the change of fat mass and HDL-C. However, there were moderate correlations between fat, especially trunk fat, and TC level. However, this result is limited by the small study population and additional research in a larger population of patients is required to confirm our findings.

Our study involved only a small number of patients from a single hospital. In subgroup analysis, the lipid indices of the obese group did not change significantly, even if the change in their lipid indices was larger than those of the non-obese group.

## Conclusion

We demonstrated that laparoscopic gastrectomy resulted in a reduction in body weight, fat mass, LBM, and BMC in both non-obese and obese groups and improvement in HDL-C in the non-obese group. The fat loss in the obese group was significantly higher and the LBM/weight ratio significantly increased, compared with those of the non-obese group. The HDL-C meaningfully increased until 12 months after gastrectomy, although the loss of fat was not significant between 6 months and 12 months after gastrectomy. The TC level is moderately correlated with fat, especially trunk fat. Future studies with a larger patient population and longer follow-up are required to confirm our findings.

## Abbreviations

BMC: Bone mineral composition
BMI: Body mass index
HDL-C: High-density lipoprotein cholesterol
LBM: Lean body mass
LDL-C: Low-density lipoprotein cholesterol
TC: Total cholesterol
TG: Triglyceride
